# Norwegian Physicians' Knowledge of and Opinions about Evidence-Based Medicine: Cross-Sectional Study

**DOI:** 10.1371/journal.pone.0007828

**Published:** 2009-11-13

**Authors:** Lidziya Vanahel Ulvenes, Olaf Aasland, Magne Nylenna, Ivar Sønbø Kristiansen

**Affiliations:** 1 Norwegian Directorate of Health, Oslo, Norway; 2 The Research Institute, The Norwegian Medical Association, Oslo, Norway; 3 Institute of Health Management and Health Economics, University of Oslo, Oslo, Norway; 4 The Norwegian Electronic Health Library, The Norwegian Knowledge Centre for the Health Services, Oslo, Norway; 5 Department of Public Health and Clinical Practice, Norwegian University of Science and Technology, Trondheim, Norway; 6 Department of General Practice and Community Medicine, University of Oslo, Oslo, Norway; 7 Institute of Public Health, University of Southern Denmark at Odense, Odense, Denmark; Universidad Peruana Cayetano Heredia, Peru

## Abstract

**Objective:**

To answer five research questions: Do Norwegian physicians know about the three important aspects of EBM? Do they use EBM methods in their clinical practice? What are their attitudes towards EBM? Has EBM in their opinion changed medical practice during the last 10 years? Do they use EBM based information sources?

**Design:**

Cross sectional survey in 2006.

**Setting:**

Norway.

**Participants:**

966 doctors who responded to a questionnaire (70% response rate).

**Results:**

In total 87% of the physicians mentioned the use of randomised clinical trials as a key aspect of EBM, while 53% of them mentioned use of clinical expertise and only 19% patients' values. 40% of the respondents reported that their practice had always been evidence-based. Many respondents experienced difficulties in using EBM principles in their clinical practice because of lack of time and difficulties in searching EBM based literature. 80% agreed that EBM helps physicians towards better practice and 52% that it improves patients' health. As reasons for changes in medical practice 86% of respondents mentioned medical progress, but only 39% EBM.

**Conclusions:**

The results of the study indicate that Norwegian physicians have a limited knowledge of the key aspects of EBM but a positive attitude towards the concept. They had limited experience in the practice of EBM and were rather indifferent to the impact of EBM on medical practice. For solving a patient problem, physicians would rather consult a colleague than searching evidence based resources such as the Cochrane Library.

## Introduction

Even though the use of systematic research has deep roots in the history of medicine, the concept “evidence based medicine” (EBM) was first introduced in 1991 by Gordon Guyatt [1]. In 1996 Sackett described EBM as “the conscientious, explicit, and judicious use of current best evidence in making decisions about the care of individual patients” and practicing evidence based medicine means “integrating individual clinical expertise with best available external clinical evidence from systematic research” and “the […] compassionate use of individual patients' predicaments, rights and preferences in making decisions about their care” [2]. By definition, Sackett states that EBM is the integration of three important aspects: current best evidence, clinical expertise and patients' values.

The ideological background for EBM has been credited to the Scottish epidemiologist Archie Cochrane (1908–1988) [3] who has been honoured through the naming of evidence-based medical research centres — Cochrane Centres — and an international organization, the Cochrane Collaboration. EBM has rapidly gained international attention and acceptance. Countless activities are denoted evidence-based and it may be hard to read a medical journal without coming across the term EBM. While Medline/PubMed did not contain the term EBM until 1992, the total number of hits for the term in March 2009 was 34 918.

Still, EBM has been met with questions and criticism. What is really meant by EBM [4]–[9]? What is the strict scientific proof that randomised controlled trials entail less bias than other study designs [7], [10]–[12] or that they improve patients' health [10], [12]–[15]? Others see EBM as scientification of the art of medicine (“cook book medicine”) or point to the evangelical tone of some EBM advocates [11], [13], [16].

Whether physicians endorse the EBM principles and apply them in their clinical practice is still largely unknown. Several studies point to problems with implementing EBM in clinical practice (*e.g.* institutional culture, lack of time, lack of information resources, impediment of clinical freedom) [15], [17]–[20]. Studies from Denmark [20] and Australia [21] indicate that textbooks and colleagues are consulted more often than the Cochrane Library.

With this background we designed a study to elucidate Norwegian physicians' opinions and experiences with EBM. Norway has a population of 4.8 million and is number two worldwide in terms of health care expenditure per capita [22]. We specifically aimed to address the following research questions:

- Do Norwegian physicians know the three key aspects of EBM?
- Do they practice EBM methods in their clinical practice?
- What are their attitudes to EBM?
- Has EBM in their opinion changed medical practice?
- What are important information sources in clinical practice?

## Materials and Methods

Data for this study stems from the so called Reference Panel of the Norwegian Physician Survey, approximately 1400 doctors of all specialties and work situations who receive a postal questionnaire from The Research Institute of The Norwegian Medical Association every other year. The questionnaires contain both questions that are repeated every time, and questions that change from one round to the next, often to meet the need of external collaborators. In 2006, a 15-page questionnaire was sent to the 1,400 professionally active panel members. It included seven composite questions about EBM with a total of 27 items. These items were sent to several colleagues in Norway and the UK for pilot testing. Most of these 27 items were formulated as statements about which respondents could indicate their position on a five point Likert scale from 1 (completely disagree) to 5 (completely agree).

The 44 officially recognized medical specialties in Norway were divided into eight groups (Table 1). Doctors in training were categorized according to their future specialty.

**Table 1:** Background data on the respondents compared with the total Norwegian doctor work-force in 2006.

Category	Subcategory	Respondents (N = 966) Percent with 95% CI	All active doctors in 2006 percent (n)
Gender (%)	Females (311)	32.2 (29.3–35.3)	33.5 (4714)
	Males (652)	67.5 (64.4–70.4)	66.5 (9364)
	Data missing (3)		
Mean age (years) in 2006	All (953)	48.8 (48.2–49.4)	49.2(14078)
	Females (305)	44.9 (43.9–45.9)	45.6 (4714)
	Males (648)	50.6 (49.9–51.3)	51.0 (9364)
	Data missing (1)		
Specialty in 2006 (%)1)	General practice (248)2)	25.7 (23.0–28.6)	25.5 (3583)
	Laboratory medicine (76)3)	7.9 (6.3–9.8)	7.9 (1106)
	Internal medicine (256)4)	26.5 (23.8–29.4)	24.5 (3447)
	Surgical disciplines (97)5)	10.0 (8.3–12.2)	12.1 (1704)
	Anaesthesiology (40)	4.1 (3.0–5.7)	5.0 (701)
	Gynaecology (37)	3.8 (2.8–5.3)	4.1 (583)
	Psychiatry (121)6)	**12.5 (10.5–14.8)**	**10.2 (1442)**
	Public health (44)7)	**4.6 (3.4–6.1)**	**6.7 (943)**
	Data missing (47)	4.9 ( 3.6–6.5)	4.0 (569)

Bold characters indicate significant differences (95% confidence intervals do not include the percentages for all doctors).

1) lncludes specialists in training.

2) includes some general practitioners who are not GP-specialists and not in training.

3) includes nuclear medicine, immunology, physiology, chemistry, neurophysiology, genetics, medical microbiology, radiology, pathology, neuropathology, pharmacology.

4) includes paediatrics, rehabilitation medicine, dermato- venerology, general internal medicine, haematology, endocrinology, gastroenterology, geriatrics, cardiology, infectious diseases, pulmonology, nephrology, neurology, rheumatology, oncology, ophthalmology, communicable diseases.

5) includes general surgery, paediatric surgery, gastrointestinal surgery, orthopaedic surgery, thorax surgery, urology, maxillofacial surgery, neurosurgery, oto-rhino-laryngology, plastic surgery.

6) includes child- and adolescence psychiatry.

7) includes occupational health.

Frequency tables and cross tables between single response variables and various groups of doctors with *χ^2^*-test were primarily used in this presentation. Violin-plots, a modification of box-plots where the density of the data is also shown (Hintze, J 2008. NCSS, NCSS LLC, Kaysville, Utah, USA www.ncss.com), are used to show various sources of information in clinical practice. We also used principal component analysis for a more comprehensive description of the doctors' attitudes towards EBM, and ANOVAs of group differences on the principal components (Table 2).

**Table 2:** Three principal components of attitudes towards EBM.

	Indifferent	Positive	Negative
It is difficult to use EBM principles in a busy clinical practice	.73		
It is difficult to search for EBM based information in a busy clinical practice	.72		
EBM focuses on the “average patient”	.68		
EBM ignores patients' values	.67		
EBM helps physicians towards better practice		.71	
EBM improves patients' health		.71	
Cochrane reviews are the most reliable		.63	
EBM is in favour of Health Care Authorities			.68
EBM is against the pharmaceutical industry			.68
EBM is a cost containment tool			.62
EBM restrains the development of high tech medicine			.51

Varimax rotated. Only loadings over 0.4 are shown.

## Results

In total 976 out of the 1,400 physicians (70%) returned a questionnaire, of which 10 were unusable. Among non-respondents, 50 explicitly stated that they did not wish to participate while 374 did not return the questionnaire despite one reminder. The respondents were quite representative of all Norwegian doctors with regard to gender, age and specialty, with slightly more psychiatrists and slightly less public health specialists (Table 1).

### Key aspects and practice of EBM

87% of the respondents agreed (score 4 or 5 on the Likert scale) that “medicine based on randomised trials” was an important aspect of EBM. Similarly, 84% indicated this for “Use of best evidence in clinical practice”, 77% for “Independent systematic review”, 53% for “Use of physician's expertise” and 19% for “Use of patients' values”.

31% of the respondents had participated in EBM courses, 51% would like to do this while 15% reported no such interest. 40% considered their practice to always have been evidence based, while 23% had changed their practice towards more EBM. The remaining 19% did not accept the distinction between evidence based and other types of medical practice.

### Attitudes towards EBM

80% of the respondents agreed that “EBM helps physicians towards better practice” and 52% agreed that it “improves patients' health” (Table 3). On the other hand, 54% indicated that it is difficult to search for evidence in a busy practice. For most of the attitude questions, including the statements about EBM and health authorities, pharmaceutical industry, cost containment and patient values, the majority neither strongly agreed nor disagreed.

**Table 3:** Frequency distributions on ten statements about EBM.

	n	1	2	3	4	5
EBM helps physicians towards better practice	943	0.1	1.9	18.2	48.8	31.0
EBM is in favour of Health Care Authorities	940	5.3	20.9	42.4	24.1	7.2
EBM is against the pharmaceutical industry	939	13.0	34.7	40.3	9.6	2.4
EBM restrains the development of high tech medicine	935	24.2	40.5	27.7	6.2	1.4
EBM is a cost containment tool	939	8.0	19.7	37.9	29.1	5.3
EBM improves patients' health	942	1.6	9.2	37.4	42.4	9.4
It is difficult to use EBM principles in a busy clinical practice	943	11.8	30.6	34.6	19.7	3.3
It is difficult to search for EBM based information in a busy clinical practice	940	3.7	13.8	28.9	37.7	15.9
EBM ignores patients' values	936	6.0	19.2	47.4	22.3	5.0
EBM focuses on the “average patient”	943	10.2	30.5	35.6	20.1	3.5

Likert scale from 1 (strongly disagree) to 5 (strongly agree). Percent.

**Figure 1:**
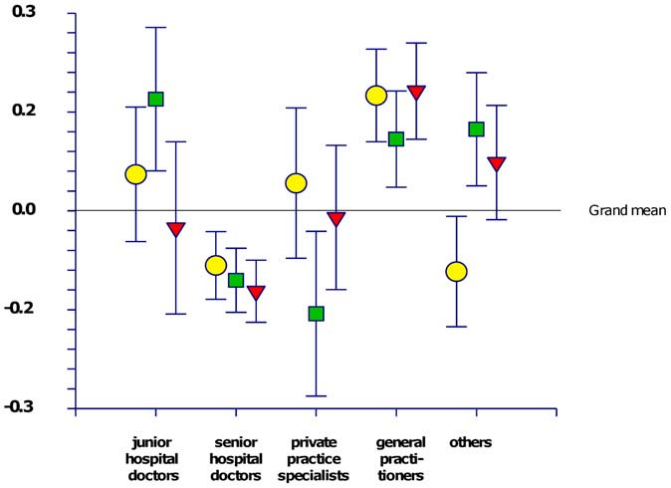
Group differences in the three identified principal components of attitudes towards EBM. Means with 95% confidence intervals. Circle – Indifferent. Square – Positive. Triangle – Negative.

For a closer analysis of attitudes towards EBM the 10 statements in Table 3 plus the statement “Cochrane reviews are the most reliable” were entered into a principal component analysis yielding three components with eigenvalue over 1, as shown in Table 2. We named the components indifferent, positive and negative, and Figure 1 shows how the different categories of doctors in the study differ with regard to these three components. The most positive group is the junior hospital doctors, who score significantly higher on “positive” than their senior colleagues. The least positive were the private specialist practitioners. Interestingly, the general practitioners score significantly higher than the grand mean on all three composite variables.

### Reason for change in medical practice

When asked about reasons for change in medical practice during the last ten years, 86% pointed to medical progress, 62% consumerism, 51% non-medical managers, 47% pointed to budget constraints and commercial interests, while 39% reported EBM.

### Information sources in clinical practice

**Figure 2:**
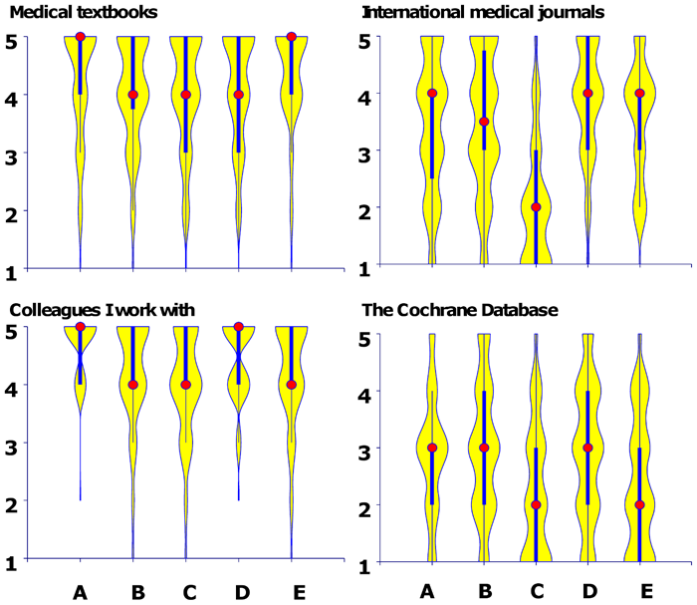
Violin plots (box plots) of responses to the use of some of the information sources in medical practice by job category. The red dots represent medians, the thick blue vertical lines the interquartile range and the yellow areas the general distribution of responses. The question was: “If you need information for the treatment of a patient, which source would you use?” Likert scale from 1 (never) to 5 (often). A - Junior hospital doctors. B - Senior hospital doctors. C - General practitioners. D - Private practice specialists. E - Other doctors.

The most frequently reported information sources (score 4 or 5) for making decisions about individual patients' health were “colleagues” (86%), “other specialists one trusts” (78%) and “medical textbooks” (76%). In total 56% and 52% reported “Medline/Pubmed” and “International medical journals”, respectively, while 27% indicated “the Cochrane database”. While the use of textbooks and colleagues varied little across physician groups, there was considerable variation with respect to international medical journals and the Cochrane Library (Figure 2).

### Physicians in surgical specialties

Few significant differences were observed between physicians in surgical specialties and other groups with regard to their age and specialty, but female surgeons were significantly underrepresented in this group (16% *versus* 34% respectively, *p*<0,000). Compared to other specialties fewer physicians in surgical specialties reported “Use of best evidence in clinical practice” (74% *versus* 86%, *p* = 0,003) and “Independent systematic review” (66% versus 79%, *p* = 0,021) as important aspects of EBM. They also seem to use other sources of information in making their clinical decisions. While 72% of them indicated international medical journals and 70% - PubMed compared to only 49% (*p*<0,000) and 53% (*p* = 0,011) of physicians in other specialties, they did not differ significantly from the rest of respondents in their low use of Cochrane Library.

### Physicians in training

Every second physician in training were a female compared to only every third among the rest of respondents (*p*<0,000), and the great majority was under 45 years old (85% *versus* 24%, *p*<0,000). Compared to other respondents fewer physicians in training reported “Use of physician's expertise” (39% *versus* 54%, *p* = 0,033) and more of them indicated “medical practice based on randomised controlled trials” (94% *versus* 86%, *p* = 0,035) as important aspects of EBM. Almost all physicians in training used their colleagues as a source of information (99% *versus* 85%, *p*<0,000) and near half of them reported that they used Cochrane Library “more or less” (score 3 at 5-point Lickert scale) (45% *versus* 27%, *p* = 0,028).

## Discussion

The results of this study indicate that Norwegian physicians have positive attitudes to EBM although their knowledge of all of its three main components (research evidence, physician expertise and patient values) is limited. They do not consider EBM to be an important factor for change in medical practice, and few have attended EBM courses or use the Cochrane database.

Our results should be viewed in the context of the study limitations. First, the sample may not be entirely representative of Norwegian physicians, even though selection bias was limited (Table 1). Second, the respondents may have responded strategically for personal reasons. Third, we did not define the term “EBM course”, and the responses here should be interpreted cautiously. Despite these limitations, however, the study results may offer interesting insight because the response rate was fairly high and most of the items relatively simple to understand.

Even though EBM “is on everyone's lips”, relatively few studies have been published about the knowledge of and attitudes toward the concept [10], [17]–[21], [23], [24]. Our findings are mainly in line with other studies. Even though we did not focus on barriers to the use of EBM, the results indicate that one-fourth found it difficult to implement EBM in busy practices. This is similar to findings in other countries where several barriers to implementation of EBM into clinical practice were reported. McAlister [19] showed that less than half of Canadian physicians felt confident in literature search. Among disincentives to using critical appraisal of literature 49% of Australasian physicians [24] reported inability to recall specific appraisal criteria and 35% inability to record the results of appraisal for future use. Lack of training and skills of critical appraisal of literature was reported by Green [18] and Hadley [17]. Like in our study, lack of time is often a barrier for EBM. In Australia and new Zealand between 60% and 90% of the physicians have this experience [21], [24]. Lack of relevant evidence was reported by 26% of Canadian physicians [19]. Lack of knowledge about EBM related sources of information among physicians was reported by Veness [21] where just over half of respondents were aware of the Cochrane Library but did not use it. The principles of practicing EBM by a five-step model [14] have also been considered a challenge in implementation [15]. When requiring systematic reviews or meta-analysis in order to answer a clinical question, Elstein claims that “…EBM moves from the desk of the practicing clinician to that of the academic researcher…“ [25]. Also, our study confirms previous findings of low utilization of typical EBM information sources and frequent use of traditional sources [19]–[21], [23], [24]. Scott [24], Veness [21] and Oliveri [20] found that only 17 to 20% did so. Among 85% of those who claimed always to practice EBM, near half had never used the Cochrane Library [20]. In Canada [19] only 5% used the Cochrane Library on a regular basis, while 93% used clinical experience and over 60% used their colleagues as their main sources for information in making clinical decisions. We do not know about other countries, but the Cochrane database and other EBM resources have been available free of charge to health personnel in Norway since 2002 and the low use of these sources seems a bit surprising.

Most of the findings in this study probably speak for themselves, but some of them are noteworthy: First, EBM has repeatedly been defined as an enterprise based on best evidence, physician expertise and patient values [2]. In practice, physicians seem to associate EBM with research evidence, and less so with expertise and particularly little with patient values. This is not surprising, however, given the fact that the latter two aspects are vague and little pursued by EBM advocates. The lack of knowledge about EBM and difficulties in implementing it in clinical practice could explain the physicians' indifference, although the term EBM is much used and warmly embraced by many key persons in health care. Relatively few have attended EBM courses, and few see EBM as a key factor for change in medical practice.

The findings of this study may provide messages to many of us. Advocates of EBM should be careful in overvaluing the impact of EBM. Those involved with medical education should realize that they still have some way to go in teaching research principles. Sir David Weatherall proclaimed that “EBM came as a gift form the Gods” [26]. Apparently, not all physicians have received it yet.
